# An Analysis of the Infections and Determination of Empiric Antibiotic Therapy in Cats and Dogs with Cancer-Associated Infections

**DOI:** 10.3390/antibiotics10060700

**Published:** 2021-06-11

**Authors:** Katie Curran, Haley Leeper, Kathy O′Reilly, Joelle Jacob, Luiz E. Bermudez

**Affiliations:** 1 Department of Clinical Sciences, Carlson College of Veterinary Medicine, Oregon State University, Corvallis, OR 97331, USA; katie.curran@oregonstate.edu (K.C.); haley.leeper@oregonstate.edu (H.L.); 2 Department of Biomedical Sciences, Carlson College of Veterinary Medicine, Oregon State University, Corvallis, OR 97331, USA; Kathy.oreilly@oregonstate.edu; 3 Oregon Veterinary Diagnostic Laboratory, Carlson College of Veterinary Medicine, Oregon State University, Corvallis, OR 97331, USA; joelle.jacob@oregonstate.edu; 4 Department of Microbiology, College of Sciences, Carlson College of Veterinary Medicine, Oregon State University, Corvallis, OR 97331, USA

**Keywords:** antibiotics, infection, patients, cancer, treatment, empiric, dogs, cats

## Abstract

Cancer patients commonly develop infectious complications over the course of the disease. One thousand patients receiving treatment for an oncologic disease at a single veterinary teaching hospital were retrospectively reviewed for concurrent infections. A total of 153 confirmed bacterial infections were identified, 82 of which were abscesses or wounds, 13 of which were respiratory infections, 3 of which were ear infections, and 55 of which were urinary tract infections. It was observed that the majority of the infections were caused by bacteria that are normally associated with that specific site location. *Escherichia coli* was the most common pathogen linked to infections in general, but *Staphylococcus pseudintermedius* was a frequently identified pathogen associated with wound infections. The susceptibility to diverse antimicrobials varied with the site of infection. Eleven cases (7.1%) were caused by opportunistic infections of the site, and *E. coli* and *Pseudomonas aeruginosa* were the pathogens isolated. Those bacteria were resistant to many antibiotics but showed susceptibility to aminoglycosides, imipenem, quinolones, and polymyxin B. In conclusion, veterinary patients with cancer or those under treatment for tumors develop infections by commonly encountered bacteria in the different sites of the body, with a susceptibility to antibiotics that is not out of line from what is expected. A small subset of cases developed opportunistic infections, with microbes that were more resistant to many classes of antibiotics.

## 1. Introduction

Infectious diseases are commonly seen in patients with cancer, both in humans [1,2] and in animals, although few studies of the latter have been reported [3]. The association between cancer and infection has many physio-pathological explanations which vary from the invasion of a tumor, many times leading to an obstruction of the lumen of the organ, such as the obstruction of the urinary tract or the airways, to infections secondary to the immunosuppression of the host, caused either by the tumor itself (such as leukemia) or induced by the chemotherapy usually used to control tumor growth [4,5].

The etiology of the infections associated with cancer is often associated with microorganisms that commonly colonize body sites, with many of the microorganisms receiving the denomination “sometime pathogens” such as *Bacteroides* sp. or *Staphylococcus* sp. Meanwhile, other microorganisms take advantage of the impairment of the host’s immune defenses resulting from the tumor itself or the employment of cytotoxic therapy to cause opportunistic infections [6]. Bacteria such as *Pseudomonas aeruginosa*, *Klebsiella* sp., *Enterobacter* sp., and fungi [7,8,9] fall into this category. These are microorganisms that can survive in the hospital environment for prolonged periods of time (6). Hospitalized patients undergo a significant change in the oropharyngeal flora, with the acquisition of hospital-related pathogens [10]. In contrast to humans, little information exists regarding infection in cats and dogs who have tumors and are undergoing chemotherapy.

There is a consensus that the use of a high dose of glucocorticoids can lead to an increased susceptibility to infection. It is known that glucocorticosteroids affect phagocytosis and the intracellular killing of pathogens by phagocytic cells, as well as affecting the number of circulating lymphocytes [11]. In additional, cytotoxic drugs can interfere with the host’s defense; this is secondary to their effects on cell proliferation [12,13].

Veterinarians, like human doctors, face decisions about the empiric use of antibiotic therapy without much information on the bacterial agent causing the infection and the pathogen’s susceptibility to antibiotics. Studies in humans have indicated a correlation between possible infecting bacteria and diverse conditions seen in cancer patients undergoing chemotherapy, therefore facilitating antibiotic selection. Very few studies addressing the issue have been performed in animals [14].

The important pieces of information to have in mind when medicating a possible infection in a patient are the most common pathogen associated with infection at that specific body site and which antibiotic would be the most appropriate to use until the microbiological results become available.

In the current study, we reviewed a database of one thousand patients with cancer seen in a veterinary hospital and identified a number of animals that presented either with infection or fever. Approximately 15% of the animals developed infection in the course of the disease or treatment. The etiologic agent and their susceptibility to antibiotics was identified, which may help us to understand the causes of common infections and their antimicrobial susceptibility in these cancer patients. This surveillance information has a couple of advantages. One advantage is that the collected data can inform veterinarians about the antibiotics that should be effective dependent on the site of infection in patients with cancer. This in itself should prevent the excessive use of therapy that is not recommended and decrease the impact of antibiotic resistance as a consequence. The second positive effect is the knowledge that, in animals with cancer, infections are caused by microorganisms of the normal biota. Our results do not include the evaluation of the treatment or outcomes of the patients, since the survey was only microbiological.

## 2. Methods

### 2.1. Subjects

Patients (dogs and cats) seen at the Oncology Service of the Veterinary Teaching Hospital (VTH) at the Carlson College of Veterinary Medicine (CCVM) from 2013 to 2018 were retrospectively analyzed regarding bacterial infections. A total of 1000 charts were analyzed and 153 of the patients had bacterial infections. Microbiological identification and antibiotic sensitivity testing was performed at the Oregon Veterinary Diagnostic Laboratory (OVDL), which is also located at the CCVM. Data were collected from a central system and assembled by matching animals with a diagnosis of cancer, the microorganisms isolated from a specimen, the site of infection, and the antibiotic susceptibility of the isolates.

### 2.2. Surveillance and Reporting

The sites of infection, infection agents, and the susceptibility to antibiotics were identified. The information was then collated and analyzed. Only one episode of infection per site was included in the survey to avoid duplication.

### 2.3. Microbiological Methods of Identification

Microbial identification was performed by established phenotypic methods and criteria using agar plates and traditional biochemical tests, as described previously [15]. All staphylococci were tested for methicillin resistance using disk diffusion susceptibility to oxacillin/cefoxitin and/or the presence of penicillin binding protein 2 (PBP2′), as determined by latex agglutination (Denka Seiken Kit, Hardy Diagnostics, Santa Maria, CA, USA). The OVDL reports all the identified organisms and does not give any specific recommendations regarding clinical importance. Notes were provided pointing out the probability that the isolates are colonizers or contaminants.

### 2.4. Antibiotic Susceptibility Testing

The isolated bacteria underwent antibiotic susceptibility testing using Kirby–Bauer disk diffusion, and minimum inhibitory concentration (MIC) was determined for the antibiotic-resistant bacteria. All isolates that did not have an antibiotic susceptibility test were excluded from the study.

Antibiotic sensitivity testing (AST) was performed only on organisms for which interpretive criteria were available (provided by the manufacturer). The OVDL uses Kirby–Bauer disk diffusion for AST and analysis is performed using BIOMICV3 (Giles Scientific, Santa Barbara, CA, USA), which utilizes the most current CLSI (Clinical and Laboratory Standard Institute) information and is updated annually. The minimum inhibitory concentration (MIC) was reported when available. All isolates were initially tested on 17 antibiotics at standard concentrations unless otherwise noted (amikacin (AN), amoxicillin/clavulanic acid (AMC), ampicillin (AM), cefovecin (CEF), cefpodoxime (CPD), cephalotin (CF), chloramphenicol (C), clindamycin (CM), enrofloxacin (ERO), gentamicin (GM), marbofloxacin (MAR), orbifloxacin (ORB), penicillin G (P), polymyxin B (PB), tetracycline (TE), Tobramycin (TM), and Trimethoprim/sulfamethoxazole (SXT)). For *Pseudomonas* sp., imipenem (IMP) was added. Chloramphenicol was not reported for urinary tract infections. No cephalosporins were reported for enterococci. Ampicillin, penicillin, and amoxicillin were reported as “resistant” for methicillin resistant staphylococci, regardless of the disk diffusion results. If drug resistance was observed, additional panels of antibiotics were tested. Methicillin-resistant staphylococci were tested against 7 additional antibiotics (azithromycin (AZT), ceftaroline (CPT), doxycycline (D), erythromycin (E) nitrofurantoin (NF), rifampin (RA), and streptomycin (S)). Resistant Gram-negative organisms were tested against 9 additional antibiotics (AZT, carbenicillin (CB100), ciprofloxacin (CIP), ceftiofur (CTF), IMP, NF, piperacillin (PIP), telithromycin (TEL15), and ticarcillin/clavulanic acid (TCC)). Aminoglycoside-resistant enterococci were tested against high-level gentamicin (120 g) and streptomycin (300 g). For monitoring purposes, vancomycin (VA) was tested on resistant Gram-positive organisms but was not reported. The laboratory does not make antibiotic treatment recommendations.

### 2.5. Data Analysis

The sites of infection, bacterial isolate(s), and the susceptibility to antibiotics were identified from the 153 cases. The information was assembled and analyzed. Only one episode of infection was included in the survey to avoid duplication. For the analysis, we counted each patient as a separate event. In cases where two microorganisms were isolated from the same patient, it was considered one episode of infection. If the same organism was isolated more than once, the most resistant isolate was included. Descriptive analysis of the extracted data of interest was standardized. The sensitivity and specificity of the tests applied were evaluated using positive and negative controls as reference standards.

## 3. Results

### 3.1. Analysis of the Population

Among the 1000 patients screened, a total of 153 were identified as having had microbiologically confirmed infections (15.3%). The most common diagnoses were lymphoma and osteosarcoma. Thirteen patients were diagnosed with pneumonia, and bronchial alveolar lavage was performed. Eighty-two patients (53.6%) had either wound infections or abscesses as the diagnosis. In three of the patients, ear and eye infections were observed, while a urinary tract infection was suspected and confirmed in fifty-five (35.9%) of the patients.

### 3.2. Common Causes of Infection

As shown in Table 1, of eighty-two cases of wound infections and abscesses (53.6% of the cases), 26.8% were caused by *Staphylococcus pseudintermedius*, 12.1% were caused by *Escherichia coli*, and 12.1% were associated with other species of *Staphylococcus*. In 11% of the cases, *Pseudomonas aeruginosa* was isolated. Moreover, 8.5% of the cases were caused by *Streptococcus* sp., 7.3% by *Enterococcus* sp., and 6.1% by coagulase negative staphylococcus. The majority of the bacteria isolated in these cases are either commonly associated with the skin, or potentially acquired in a hospital environment.

Urinary infections were caused by *E. coli* (42.2% of the cases), *S. pseudintermedius* (23.6%), and *Enterococcus* sp. (12.7%). The percentage of *E. coli* infections is almost predictable, but one would question the presence of *S. pseudintermedius* as a possible skin contaminant.

Among the cases where BALF (Bronchial Alveolar Lavage Fluid) was performed, several were positive to *Pasteurella multocida* (38.4%) and *E. coli* (30.7%). *Enterococcus* sp., *Proteus mirabilis*, *Bordetella bronchiseptica*, and coagulase negative Staphylococcus had one case each. While *P.multocida* would be predictable as a colonizer becoming a pathogen in cats and dogs, the fact that approximately 30% of the BALF isolates emerged as *E.coli* was not expected. 

### 3.3. Susceptibility to Antibiotics

The empirical treatment of a cancer patient with an infection is usually blind until the diagnosis becomes available. As shown in Table 2, the antibiotics that showed more activity against the bacteria isolated from the respective sites are indicated. The table only includes antibiotics with more than 60% activity against the bacteria isolated.

In general, amikacin showed a very good activity in all of the different infections. When evaluating wound infections, only amikacin, imipenem, and ceftaroline showed reliable and consistent activity. For the treatment of urinary infections, aminoglycosides, third-generation cephalosporins, quinolones, and SMX/TMP demonstrated reasonable activity.

Amoxicillin is commonly used to treat infections in pets with infections and cancer. Our study demonstrates that, for the exception of a wound infection, amoxicillin plus clavulanic acid can be an effective alternative until the bacteriologic results become available.

### 3.4. Infections Caused by Opportunistic Microorganisms

Infections in patients with cancer can be associated with immunosuppression, either caused by the tumor or secondary to the therapy. We looked at the infections caused by opportunistic pathogens. A total of 11 cases were identified: three infections caused by *E. coli* (wound infections), six infections (five skin/wound infections and one urine) caused by *P. aeruginosa*, one urinary infection caused by *Citrobacter* sp., and one urinary infection caused by Enterobacter sp. Two among the *E. coli* isolates were resistant to 3rd-generation cephalosporins and quinolones. All of the *E. coli* isolates were susceptible to aminoglycosides. Both *Citrobacter* and *Enterobacter* were susceptible to all antibiotic classes, suggesting that they are not linked to the hospital environment. Among the *P. aeruginosa* isolates, all were susceptible to amikacin, tobramycin, and imipenem, and three were susceptible to marbofloxacin and polymyxin B as well. Four out of the five *Pseudomonas* isolates were resistant to 3rd-generation cephalosporins (Table 3).

### 3.5. Infections Caused by Staphylococcus sp.

Methicillin-resistant *Staphylococcus* is a significant problem in both human and veterinary medicine. Many of the *S. pseudintermidius* identified in the hospital environment are methicillin-resistant and resistant to many other antibiotics. As shown in Table 4, many of the *S. pseudintermedius* are methicillin-resistant and also resistant to many first-line antibiotics.

## 4. Discussion

Infectious diseases and cancer are commonly associated. Tumors can obstruct structures, remove natural barriers, and allow for bacterial overgrowth. In addition, both chemotherapy and the tumor itself can lead to the immunosuppression of the host’s defenses, thus facilitating infection by opportunistic pathogens [16,17].

This study examined 1000 patients seen at our facility, from which 153 bacterial infections were microbiologically confirmed. The infection sites identified in this population included the airways and lungs, eyes and ears, urine, and abscesses and wounds. In the majority of the cases, the bacteria responsible for the infection are commonly encountered at and associated with the body site involved. These organisms are usually present as colonizers of the mucosa or skin surfaces and cause clinical disease only when they are introduced to sterile tissues by a break in the integrity of the surface of the tissue. Organisms that possess virulence determinants can cause infection. In immunosuppressed patients, however, even bacteria that cannot establish an infection in a healthy host can successfully cause disease. Normal biota and organisms that can adhere to cell structures can become pathogens [18].

Cancer treatment in dogs and cats does not involve the delivery of chemotherapeutic drugs at doses that often cause pancytopenia and severe immunosuppression. The consequence of the approach is that dogs and cats under cancer treatment will rarely develop a neutropenia-associated infection or septicemia caused by opportunistic pathogens [12,13]. In fact, our results show that the majority of infections seen in those animals are caused by microorganism colonizers of the body sites. It is also important to consider that some chemotherapeutic agents can be cytotoxic or induce partial immunosuppression, even when used at doses not associated with absolute neutropenia (neutrophils <500). In the case of solid tumors, the patients generally undergo surgery as part of the therapy, although data on infections in patients with solid tumors who are not neutropenic are scarce both in humans and in animals [1]. In fact, in human reports, Staphylococcus sp. were isolated in approximately 45% of the cases, while Gram-negative bacterial infections were the diagnostic infection in 13–15% of the reports [1,16,19].

Another important consideration in this population of cancer patients is that many times they develop fever associated with tumor progression or necrosis, many times as result of transient bacteremia, which is difficult to document microbiologically. In many of these examples, the animal is placed on antibiotics, which can be linked with the colonization of body sites by opportunistic pathogens and antibiotic-resistant bacteria [20]. Additionally, fungal infections are important; they usually follow antibiotic use.

In our study, *E. coli* and *Proteus mirabilis* were isolated from bronchial alveolar lavage fluid and these are not common microorganisms isolated from the respiratory tract. The *E. coli* were resistant to many antibiotics and showed partial susceptibility to amikacin and tobramycin (Table 2 and Table 3). Besides these infections, 9 infections were caused by *P. aeruginosa*, 10 infections by *E. coli*, and 4 infections by *Enterobacter* species, whereas most of the remaining episodes of infections were caused by Gram-positive organisms, which are skin-associated bacteria. Many of the wound infections, 49 out of 82 were caused by Staphylococcus sp. The most common bacterial isolate was *S. pseudintermedius*, but coagulase negative *Staphylococcus*, *S. aureus*, and *S. schleiferi* were also common. *S. pseudintermedius* is an organism frequently isolated from the nares, ears, mouth, anus, forehead, and skin of dogs [21]. It has the ability to form biofilms in the environment and in the host [22,23,24]. *S. pseudintermedius* has also been isolated from humans and their household pets, with a fraction of them being resistant to methicillin [25]. Recently, we reported environmental *S. pseudintermidius* as a common cause of hospital-related infections in dogs [23]. Published work suggests that the mechanism of virulence in *S. pseudintermedius* may be very different from the pathogenic mechanism associated with *S. aureus* [26]. Infections of the urinary tract by *E. coli*, *Enterobacter* sp., and *Enterococcus* sp. were not surprising, as these infections are frequently observed as etiology bacteria commonly associated with urinary tract infections in dogs and cats. As shown in Table 4, many of the strains of *S. pseudintermidius*, both susceptible and resistant to methicillin, can show susceptibility to rifampin in vitro. As in the case of *S. aureus* infection, one should be aware that the use of rifampin alone for the treatment of such infections would very likely result in the development of resistance. A recent study identified mutations in the rpoB gene in *S. pseudintermedius* soon after the initiation of the rifampin administration [27].

One of the motivations to investigate infections in cancer patients is to be able to determine the range of susceptibilities to antibiotics among the pathogens. In many cases, cancer-associated infections require the initiation of therapy without any potential clue regarding the pathogen causing the infection. In addition, exposing animals to continuous stress may have an impact as a cause of infection in animals [28]. Our study demonstrates that infections of the respiratory tract related to *Pasteurella* would respond quite well to the antibiotics normally used to treat *Pasteurella* infection, such as amoxicillin/clavulanic acid or quinolones. In contrast, if the respiratory infection is caused by *E.coli*, the chances are that these bacteria would be multi-resistant, requiring treatment with antibiotics of the class of aminoglycosides. Abscesses were usually caused by antibiotic susceptible bacteria, in contrast to bacteria isolated from wounds, which showed significant resistance to the available antibiotics. For example, *S. pseudintermedius* were only susceptible to amikacin and, in 64% of the cases, susceptible to gentamicin. It is known that the number of cases of multi-resistant *S. pseudintermedius*, including methicillin resistance, in veterinary hospitals is increasing significantly [21,23], but the other pathogens associated with wound infections were also multi-resistant to antibiotics. The only exception to susceptibility was the activity of the 5th-generation cephalosporin, ceftaroline, which was very active against all of the *Enterococcus* spp. Since ceftaroline was developed to have activity against MRSA and vancomycin-resistant enterococcus, this adds to the prescription indications [29].

Patients with a cancer diagnosis have increased chances of developing infections causes by opportunistic pathogens. Both the tumor by itself and the immunosuppression caused by therapy are linked to the association. Although opportunistic pathogens do not represent the majority of the cases with microbiological evidence of infection, Gram-negative bacilli were isolated from such infections. The results in our patient collection support the idea that infections linked to host immunosuppression are not as common in veterinary medicine when compared to human oncology patients. We observed a small percent of patients in this category, and the susceptibility to antibiotics by the causing microbes was in many cases broad. Only a few organisms were resistant to 3rd-generation cephalosporins and quinolones. The observation is important for veterinarians, since prevention of infection can be achieved by a careful examination of the patient’s biota before the beginning of anti-cancer therapy. The approach should have an impact on the use of antibiotics.

The use of antibiotics in cancer patients with fever, most of the time, involves broad spectrum antibiotic therapy. The knowledge of the common microorganisms causing the infections by site should limit the use of antibiotics to narrow spectrum drugs. As a note of caution, antibiotic susceptibility tests in vitro represent a solid piece of information that can help the prescriber to decide on the treatment. However, other important aspects, such as abscess drainage or pharmacokinetics and chemical aspects of certain antimicrobials, should be always considered.

As a general conclusion, the majority of the infections observed in dogs and cats with the diagnosis of cancer, and many times undergoing treatment for the disease, are caused by bacteria which normally cause infection in the specific site of the body involved. Infections caused by opportunistic bacteria, although observed, were not common, except in the specific cases of wound infections, many of which were acquired in the hospital.

The results reported in our study could offer guidance to veterinary oncologists treating their patients and support the idea that infections linked to host immunosuppression are not as common in veterinary medicine as compared with human oncology patients. The limitation on the aggressiveness of the anti-tumor therapy dictates the sort of infections in the patient population.

## Figures and Tables

**Table 1:** Causes of infections by site in dog and cat patients with a cancer diagnosis.

Infection Site	Bacteria	Number of Cases
Respiratory (BALF)	*Enterococcus* sp.	1
	*Escherichia coli*	4
	*Pasteurella multocida*	5
	*Proteus mirabilis*	1
	*Staphylococcus* coagulase negative	1
	*Bordetella bronchiseptica*	1
	Total number of cases	13
Abscess/Wound Swabs	*Pseudomonas aeruginosa*	9
	*Staphylococcus pseudintermedius*	22
	*Vagococcus*	1
	*Enterococcus* sp.	6
	*Escherichia coli*	10
	*Pasteurella canis*	1
	*Pasteurella stomatis*	1
	*Proteus vulgaris*	1
	*Staphylococcus aureus*	3
	*Staphylococcus schleiferi*	2
	*Staphylococcus* sp.	10
	*Staphylococcus* coagulase negative	5
	*Staphylococcus* β-hemolytic	7
	*Enterobacter* sp.	4
	Total number of cases	82
Ear/Eye Swabs	*Escherichia coli*	1
	*Streptococcus* β-hemolytic	1
	*Staphylococcus* coagulase negative	1
	Total number of cases	3
Urine	*Enterobacter* sp.	1
	*Proteus vulgaris*	2
	*Staphylococcus* sp.	1
	*Klebsiella* sp.	1
	*Staphylococcus pseudintermedius*	13
	*Enterococcus* sp.	7
	*Pseudomonas aeruginosa*	2
	*Citrobacter* sp.	2
	*Escherichia coli*	26
	Total number of cases	55

**Table 2:** Antibiotics with activity against infections isolated from several sites.

Infection Site	Number of Cases	Most Common Pathogens	Antibiotics with Activity (% Susceptible Bacteria)	Notes
Ear/Eye	3	*Escherichia coli, Streptococcus* β-hemolytic, *Staphylococcus* coagulase negative	Amikacin (66%), gentamicin (100%), tobramycin (66%), amoxicillin/clav (100%), ampicillin (100%), cefovecin (100%), cefpodoxime (100%), cephalothin (100%) enrofloxacin (50%), marbofloxacin (100%), SMX/TMP (100%), tetracycline (66%)	All the isolates were resistant to orbifloxacin
(BALF)	13	*Escherichia coli*, *Pasteurella multocida* (9 cases)	Amikacin (71.4%), tobramycin (71.4), amoxicillin/clav (83.3%), cefovecin (100%), enrofloxacin (83.3%), TMP-SMX (83.3%)	
Abscess	4	*Vagococcus, Escherichia coli, Staphylococcus aureus*	Amikacin (100%), amoxicillin/clav (100%), cefovecin (100%), cefpodoxime (100%), cephalothin (75%), chloramphenicol (75%), marbofloxacin (100%), orbifloxacin (100%), enofloxacin (75), SMX/TMP (75) (*)	(*) can be inactive
Wounds Swabs	78	*Staphylococcus pseudintermedius* (27%)	Amikacin (80.7%), gentamicin (64%), cefovecin (69%), cefpodoxime (61%) enrofloxacin (67.6%), marboflaxcin (69%), SMX/TMP (61%), polymyxin B (61%), imipenem (100%), ceftaroline (100%)	
		*Escherichia coli* (12%)		
		*Staphylococcus* sp. (12%)		
		*Pseudomonas aeruginosa* (11%)		
		*Staphylococcus* β-hemolyticus (8.5%)		
		*Enterococcus* sp. (7.3%)		
Urine	55	*Escherichia coli* (47%)	Amikacin (87%), gentamicin (75%), tobramycin (75%), amoxicillin/clav (75%), cefovecin (81%), cefpodoxime (80%), ceftaroline (100%) (*) Enrofloxacin (79%), marbofloxacin (79%), orbifloxacin (73%), SMX/TMP (76%)	(*) fifth-generation cephalosporin with activity against Gram-positive bacteria
		*Staphylococcus pseudintermedius* (24%)		
		*Enterococcus* sp. (13%)		

**Table 3:** Infections caused by opportunistic bacteria (*E. coli* and *P. aeruginosa*) and their susceptibility to antibiotics.

Site of Infection	Bacteria	Susceptibility
Skin infection	*E. coli*	Amikacin, Gentamicin, Tobramycin, Polymyxin B, Enrofloxacin, Marbofloxacin, TMP/sulfa
Peritoneal swab	*E. coli*	Amikacin, Gentamicin, Tobramycin, Polymyxin B, Enrofloxacin, Marbofloxacin, TMP/sulfa
Surgical site	*E. coli*	Amikacin, Imipenem, Polymyxin B
Skin swab	*P. aeruginosa*	Amikacin, Gentamicin, Tobramycin, Marbofloxacin, Polymyxin B, Imipenem
Skin Swab	*P. aeruginosa*	Amikacin, Gentamicin, Tobramycin, Enrofloxacin, Marbofloxacin, Imipenem
Urine	*P. aeruginosa*	Amikacin, Enrofloxacin, Marbofloxacin,
Surgical swab	*P. aeruginosa*	Amikacin, Gentamicin, Tobramycin, Enrofloxacin, Marbofloxacin
Surgical swab	*P. aeruginosa*	Amikacin, Gentamicin, Tobramycin, Imipenem
Surgical swab	*P. aeruginosa*	Amikacin, Gentamicin, Tobramycin, Imipenem, Marbofloxacin

*Citrobacter* sp. and *Enterobacter* sp. isolated from two patients were susceptible to all the tested antibiotics.

**Table 4:** *Staphylococcus* sp. isolates from diverse sites in the cancer patient population.

Bacterial Species	Number	Susceptibility to Antibiotics of Isolates
*Staphylococcus* coagulase +	7	All susceptible to amikacin, TMP/SMX 4 susceptible to amoxicillin 5 susceptible to cefpodoxime
*Staphylococcus pseudintermidius*	31	16 were methicillin-resistant 27 susceptible to amikacin 19 susceptible to cefpodoxime 13 susceptible to enrofloxacin 13 susceptible to TMP/SMX 15 susceptible to rifampin
*Staphylococcus* coagulase −	7	3 were methicillin-resistant All susceptible to amikacin 5 susceptible to TMP/SMX 6 susceptible to cephalosporins 5 susceptible to enrofloxacin
*Staphylococcus aureus*	3	All susceptible to amikacin, amoxicillin 2 susceptible to enrofloxacin, cephalosporins

TMP/SMX: trimethoprim/sulfamethoxazole.

## Data Availability

All the data is available upon request from the coressponding author.
